# The spatiotemporal heterogeneity of the biophysical microenvironment during hematopoietic stem cell development: from embryo to adult

**DOI:** 10.1186/s13287-023-03464-8

**Published:** 2023-09-13

**Authors:** Guolin Shi, Pan Zhang, Xi Zhang, Jing Li, Xinmin Zheng, Jinxiao Yan, Nu Zhang, Hui Yang

**Affiliations:** 1 School of Life Sciences, Northwestern Polytechnical University, Xi’an, Shaanxi China; 2 Engineering Research Center of Chinese Ministry of Education for Biological Diagnosis, Treatment and Protection Technology and Equipment, Xi’an, Shaanxi China; 3 Research Center of Special Environmental Biomechanics & Medical Engineering, Northwestern Polytechnical University, Xi’an, Shaanxi China; 4 School of Food Science and Engineering, Shaanxi University of Science & Technology, Xi’an, China; 5 Shaanxi Key Laboratory of Brain Disorders & Institute of Basic and Translational Medicine, Xi’an Medical University, Xi’an, China

**Keywords:** Spatiotemporal heterogeneity, Biophysical microenvironment, Hematopoietic stem cells, Fate regulation

## Abstract

Hematopoietic stem cells (HSCs) with the ability to self-renew and differentiate are responsible for maintaining the supply of all types of blood cells. The complex and delicate microenvironment surrounding HSCs is called the HSC niche and can provide physical, chemical, and biological stimuli to regulate the survival, maintenance, proliferation, and differentiation of HSCs. Currently, the exploration of the biophysical regulation of HSCs remains in its infancy. There is evidence that HSCs are susceptible to biophysical stimuli, suggesting that the construction of engineered niche biophysical microenvironments is a promising way to regulate the fate of HSCs in vitro and ultimately contribute to clinical applications. In this review, we introduced the spatiotemporal heterogeneous biophysical microenvironment during HSC development, homeostasis, and malignancy. Furthermore, we illustrated how these biophysical cues contribute to HSC behaviors, as well as the possible mechanotransduction mechanisms from the extracellular microenvironment into cells. Comprehending the important functions of these biophysical regulatory factors will provide novel approaches to resolve clinical problems.

## Introduction

Hematopoietic stem cells (HSCs) are heterogeneous cells at the top of the hematopoietic system that exhibit self-renewal and differentiation abilities [1, 2]. The hematopoietic system uses billions of fresh cells every day to update the blood and immune cells in the body [3]. HSCs maintain hematopoietic homeostasis through self-renewal, maturation, apoptosis, resting mode, and trafficking in vivo, which are highly complex and controlled physiological features [4]. Since the 1960s, HSCs have been used in the clinical treatment of patients with hematological diseases such as leukemia and lymphoma [5]. The main sources of HSCs for clinical applications are cord blood (CB), adult bone marrow (BM), and mobilized peripheral blood stem cells. However, because of the low availability of matching donors and the number of HSCs per transplant, the supply of HSCs is very limited [6]. The clinical bottleneck of hematopoietic stem cell transplantation (HSCT) is how to efficiently proliferate functional HSCs in vitro. This challenge may be addressed by studying the growth environment of HSCs in vivo.

HSC niches are highly specialized microenvironments that provide HSCs with all the signals that regulate HSC survival, maintenance, proliferation, and differentiation [7]. Since the HSC niche hypothesis was proposed, researchers have continued to discover new cells, factors, and other parameters that play a role in these niches [8, 9]. Efforts have been made to understand the heterogeneity of these niches at different stages of development, homeostasis, and malignancy [10], enriching the complexity of niche regulation. The microenvironment surrounding HSCs can provide physical, chemical, and biological stimuli that modulate HSC activity and fate alone or in combination. Nevertheless, the exploration of the biophysical regulation of HSCs remains in its infancy [11–13]. This review discusses the heterogeneity and the important roles of these biophysical microenvironments in HSC fate regulation, which have led to exciting new ideas for the design of artificial niches for HSC engineering and clinical applications.

## Complexity of HSC niches

### Spatiotemporal heterogeneous niches during HSC development, homeostasis, and malignancy

**Fig. 1:**
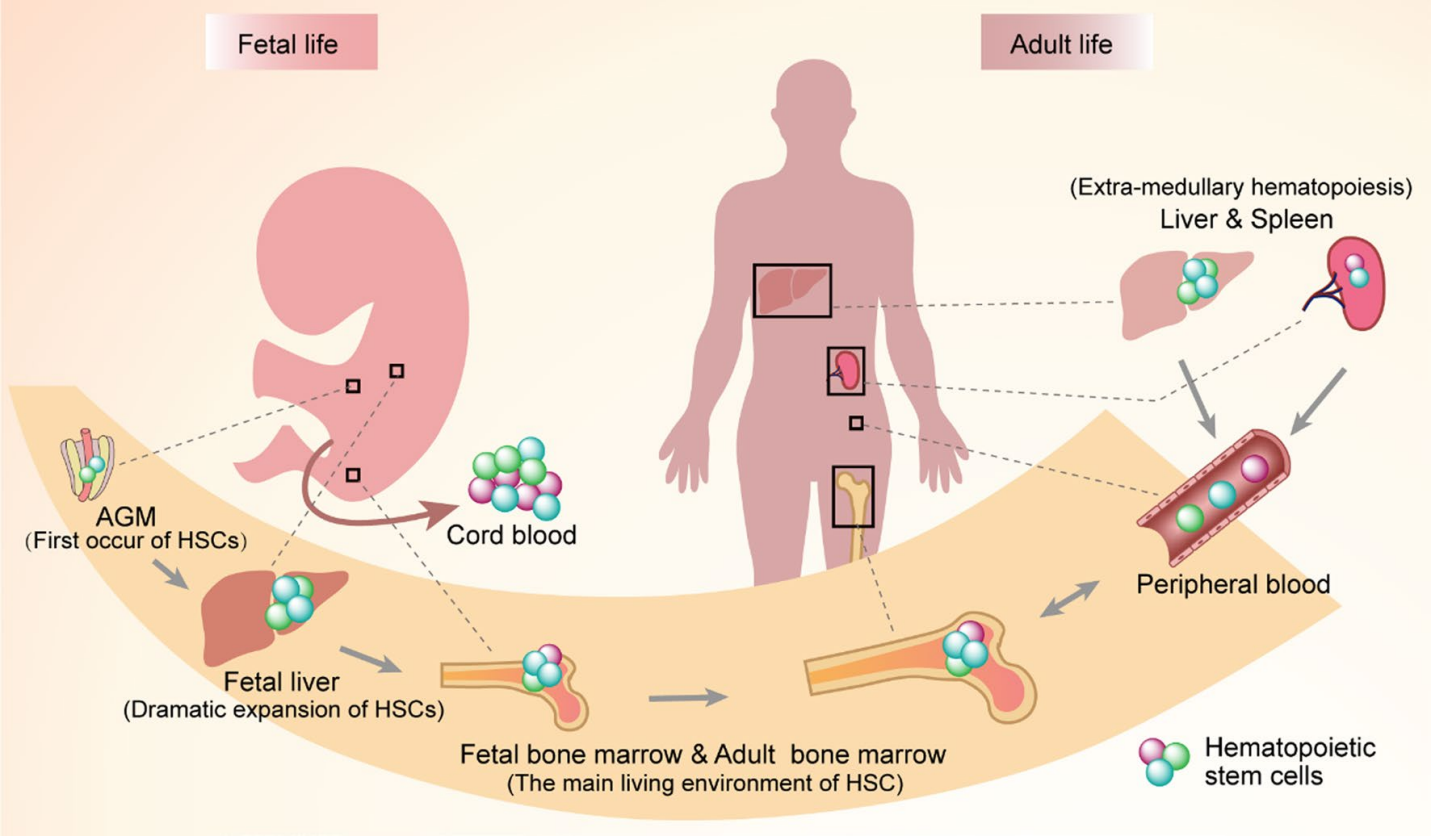
Anatomical location of hematopoietic stem progenitor cells (HSPC) niches changes with space and time. HSPCs are found in many organs in the body across a lifetime. AGM, aorta-gonad-mesonephros

The abilities of HSC self-renewal and maintenance are strictly regulated throughout the human lifetime to maintain blood system homeostasis. Recent technological progress has shown that HSC and hematopoietic stem and progenitor cell (HSPC) groups are not discrete homogeneous populations, but rather heterogeneous populations. Evidence shows that there are HSCs with lineage bias and progenitor cells with lineage restriction in HSC niches [14]. Previous studies have drawn a single-cell transcription map of human hematopoietic stem cells from hematopoietic endothelium to birth and have found that HSC and progenitor cells can be distinguished based on their signature [2]. The HSC niche in development also shows heterogeneity. The anatomical location of HSPC niches changes with space and time (Fig. 1).

Primitive hematopoiesis begins in the yolk sac, and definitive hematopoiesis occurs in the aorta-gonad-mesonephros (AGM) region [15]. HSCs then undergo active expansion and specification in the fetal liver [16]. Afterward, HSCs emigrate from the fetal liver to the fetal spleen [17]. Finally, stromal cell-derived factor 1 generated by BM stromal cells induces the expression of CXCR4 from HSC, which mediates HSC reside into the BM [18].

Throughout adult life, HSCs are maintained and regulated in BM niches. According to different anatomical positions, niches close to the endosteum are currently defined as endothelial niches [19], while niches close to the BM sinusoids [20] or arterioles [21] are defined as perivascular niches. Obviously, each of these BM niches is produced by a variety of cell types and the constituent cells vary greatly [22]. These different microenvironments have different functions, and the HSCs hosted in them are also heterogeneous [23]. Many recent studies have confirmed that the phenotypes and functions of HSCs are heterogeneous in different niches [24, 25]. The use of new technologies has revealed that HSCs are not a pool with unified functions, but a heterogeneous pool composed of different HSC subgroups. HSCs comprise several HSC subgroups with different immunophenotypes. These subgroups have different self-renewal and regeneration abilities, and the selectivity of lineage differentiation is also biased [26]. In addition, there is reason to believe that the heterogeneity of HSCs is closely related to the heterogeneity of HSC niches.

Generally speaking, the main site of hematopoietic activity is the BM. However, when the BM microenvironment becomes unsatisfactory, extra-medullary hematopoiesis may occur in the liver or spleen [27]. When the blood system is stable, the HSCs are in a dormant state. When infection, acute blood cell loss, chemotherapy, radiation-induced cytotoxicity, and other forms of stress and injury occur, HSCs can reversibly switch from a dormant state to an active state to restore the stable state of hematopoiesis. Once the blood system regenerates and re-establishes its stable state, the activated HSCs will re-enter their dormant state [28, 29]. HSCs in a dormant state can minimize the accumulation of DNA damage, thus preventing HSC exhaustion and BM failure [30, 31].

Alterations to the hematopoietic microenvironment upon aging might lead to diseases such as hematologic malignancies [10]. Therefore, the compositional and functional heterogeneity of niches in various anatomical sites during different developmental stages should be emphasized when identifying their special contributions to the fate of HSCs. The aging of HSCs partially contributes to the impairments of an aged hematopoietic system. Research shows that in young- and middle-aged mice, there is a stable balance between myeloid-biased, lymphoid-biased, and balanced HSC subsets [32, 33]. However, this balance was found to be broken in older mice. Myeloid-biased HSCs increased and became the main type. The self-renewal and regeneration ability of these HSCs decreased, resulting in a decline in the production of mature blood cells [24, 34]. Aging leads to the reduction in comfort of the HSC niche, thus breaking the hematopoietic homeostasis. Some studies have changed the mitochondrial membrane potential of HSC through pharmacological operations and have found that it can have beneficial effects on the function of HSC [35].

### Multiple components of HSC niches

**Fig. 2:**
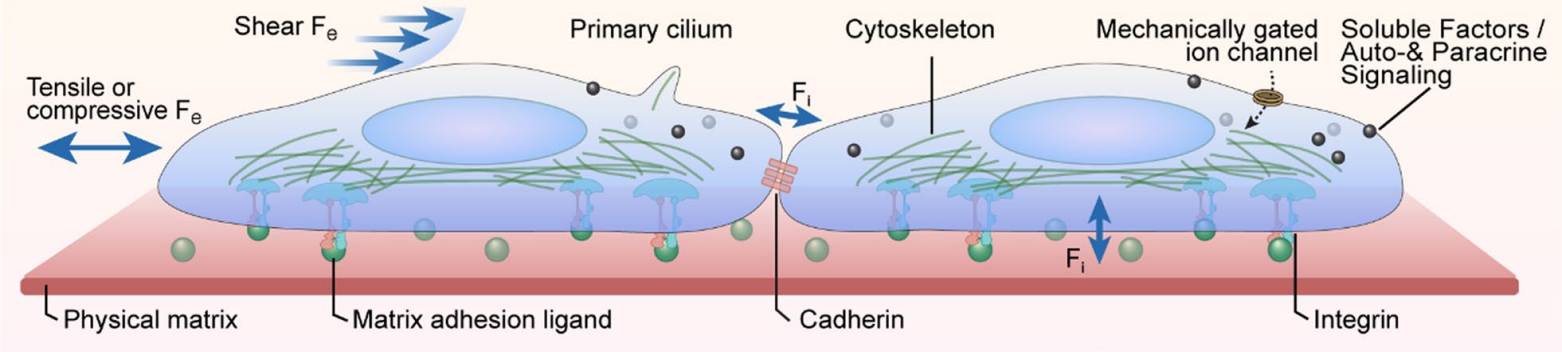
Hematopoietic stem cells (HSCs) are stimulated by the biochemical, biological, and physical parameters of the microenvironment in vivo. HSCs are subjected to biochemical and biological signals elicited by cell–cell interactions including direct contacts and communication through soluble factors as well as cell–matrix interactions. At the same time, cells are stimulated by the physical parameters of the environment. Intrinsic forces (*F*_i_) are generated intracellularly and transferred to other cells through cell–cell junctions, such as cadherin receptors, or via traction on extracellular matrix (ECM) adhesion ligands those are bound to integrin receptors. Extrinsic forces (*F*_e_) are externally applied by shear or tension and/or compression on cells, and they can be sensed by mechanically gated ion channels, changes in receptor-ligand binding, deformation of the cytoskeleton, and the primary cilium. Physical properties, for example, the elastic modulus and nanotopography of the ECM, govern how mechanical cues are transduced. The cytoskeleton generates and transfers forces from membrane proteins to intracellular structures, such as the nucleus

The communication between a variety of niche components, including niche cells, soluble components, and ECM molecular provides chemical signals regulating the fate decision of HSC. As shown in Fig. 2, the mechanosensors formed by cell–cell and cell–ECM interactions, such as adhesion receptor-ligand bonds, the cytoskeleton, mechanically gated ion channels, and primary cilia, enable HSCs to autonomously sense and react to mechanical cues in niches [36, 37]. Components of HSC niches that contribute to HSC fate regulation are shown in Table 1.

**Table 1:** Components of HSC niche

Cellular components	Extracellular matrix	Biophysical parameters
Mesenchymal stem cells	Polysaccharide	Shear stress
Osteolineage cells	Proteoglycans	Hydrostatic pressure
Adipocytes	Collagens	Circumferential strain
Endothelial cell	Laminins	Stiffness
Sympathetic nerves	Fibronectin	Viscoelasticity
Schwann cells	Osteopontin	3D architecture
Macrophages		Nanotopography
T cells		
Neutrophils		

HSC niches are composed of multiple cell types with specific functions [38]. These different types of cells either directly or indirectly support the maintenance and regulation of HSCs in HSC niches [39]. Mesenchymal stromal cells (MSCs) provide niche factors such as CXCL 12, SCF, and interleukin 7 (IL 7) and play an important role in the regulation of HSCs [40]. Osteolineage cells (osteocytes, osteoblasts, and osteoclasts) are crucial for lymphopoiesis and have been implicated in HSPC regulation [41]. Adipocytes might inhibit HSC activity, but this conclusion is still controversial [42, 43]. The endothelium can regulate HSC maintenance and the activity of perivascular cells [44]. Adrenergic nerves can regulate HSC mobilization and hematopoietic recovery [45]. Schwann cells may promote HSC quiescence through TGF-β signal conduction [46]. Macrophages may directly participate in the maintenance of HSC, and indirectly regulate the retention of HSC through niche cells [47]. Megakaryocytes can promote HSC quiescence via applying a feedback loop [48, 49]. T cells and neutrophils might direct interact with HSCs or regulate HSC behaviors via other immune and stromal cells [50, 51]. Recent studies (Table 1) have continuously enriched the known functions of each cell and factor during HSC homeostasis and niche formation. Although many studies have been conducted to elucidate the role of cells in the niche, the regulation of HSC populations remains highly complex and elusive.

The ECM is a fine and intricate network composed of macromolecules synthesized and secreted by cells to the outside that are distributed on the cell surface or between cells. Its components are mainly collagen, elastin, non-collagen glycoprotein, and aminoglycan or proteoglycan. The ECM maintains the organizational structure of cells and provides anchorage sites for cell adhesion and migration [52, 53]. A variety of ECM proteins participate in the regulation of HSCs. For example, structural proteins such as collagen, laminin, and fibronectin provide anchorage sites for cells to support HSC retention and mobilization [52]. In addition, laminins and fibronectin have also been found to affect the regulation of HSC proliferation and differentiation, and affect the implantation ability of HSC [54–56]. Osteopontin has been found to have a negative effect on HSC proliferation [57, 58]. The ECM in the BM is heterogeneous. The endothelial region is rich in osteopontin and type I collagen, and the vascular region is rich in laminin [59–61]. The changes in the ECM composition in different regions may lead to different functions of BM niches. Variations in the ECM composition may contribute to differences in biological, chemical, and biophysical factors in BM regions.

Studies have revealed that HSCs have the ability to sense external biophysical cues, such as shear stress, matrix stiffness, and matrix nanotopography [13]. These biophysical stimuli, alone or coupled with biological stimuli, can modulate the activity and fate of HSCs. HSCs convert the macroscale biophysical inputs they sense into molecular signals with chemical activity to guide cell behavior [62]. However, the mechanotransduction mechanism through which HSCs sense and react to the mechanical signals remains unknown.

## Heterogeneous biophysical microenvironment of HSCs

**Fig. 3:**
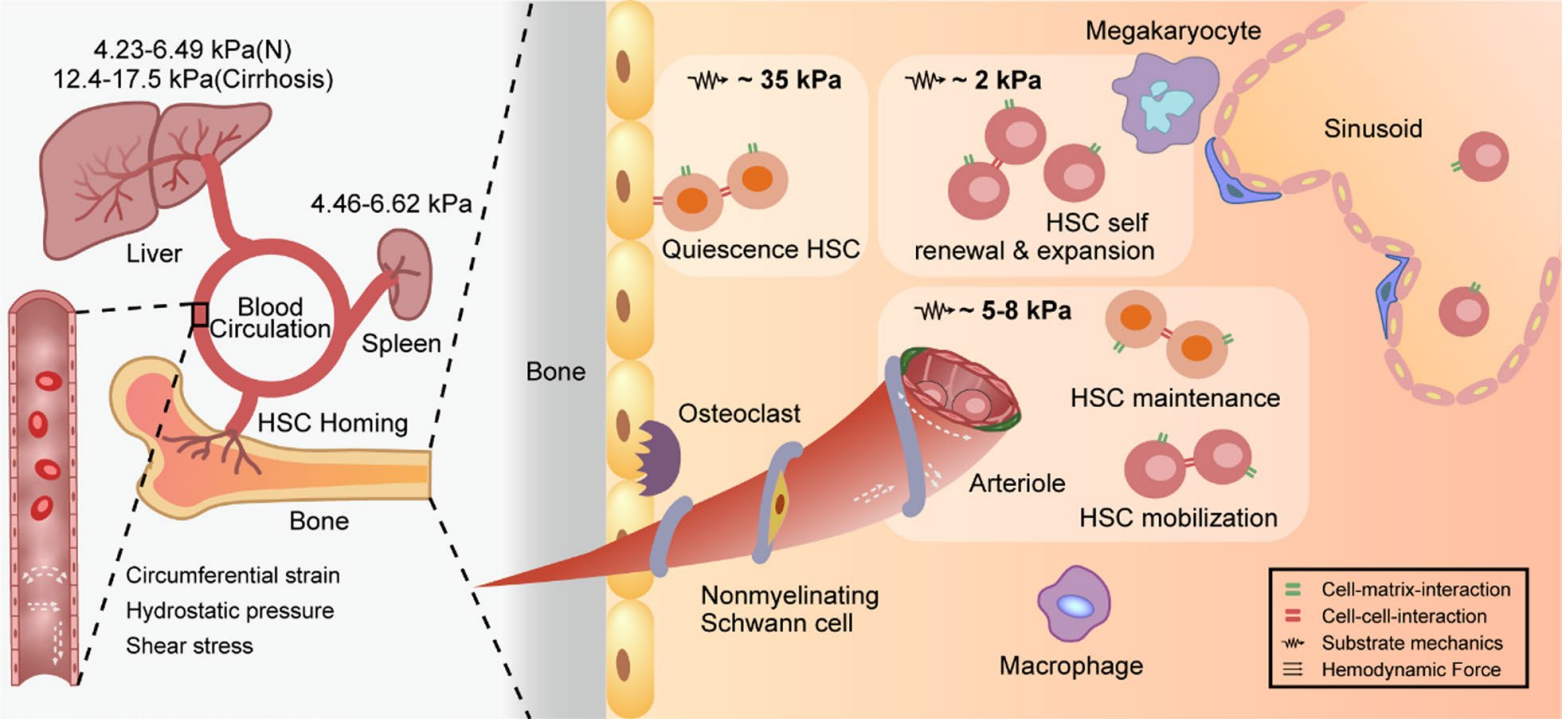
Biophysical microenvironment of heterogeneous HSC niches. Bone marrow is the primary niche for adult HSC maintenance. When the individual is under severe stress or the BM microenvironment becomes suboptimal due to pathological conditions, extra-medullary hematopoiesis (EMH) can occur, mostly in the liver or spleen

HSC maintenance and self-renewal are strictly regulated throughout the human lifetime to maintain blood system homeostasis. HSC niches are spatiotemporally heterogeneous during HSC development, homeostasis, and malignancy. In studying the specific contributions of the biophysical microenvironment to the fate of HSCs, the heterogeneity of the composition and function of niches in different anatomical sites at different developmental stages should be emphasized (Fig. 3).

### Biophysical microenvironment of embryonic HSCs

Studies have shown that hematopoietic stem cell formation, development, and regulation are dependent on blood flow [63–65]. Blood flow and hematopoietic cells occur synchronously, and research also shows that blood flow is an important regulatory factor for HSC development [66]. Blood flow passing through a vessel generates three forces: hydrostatic pressure, shear stress, and circumferential strain. Shear stress is the frictional force tangential to endothelial cells (ECs), while circumferential strain refers to the force perpendicular to the flow direction [67, 68]. At the embryonic stage, HSCs are generated by the hematopoietic endothelium in the AGM. Previous studies have shown that shear stress caused by blood flow is a necessary condition for HSC generation in this process [37, 62]. Similarly, studies in vitro have shown that shear stress affects the proliferation and differentiation of HSCs in vitro. These studies provide a new idea for the expansion of HSCs in vitro and will have a beneficial impact on the possible clinical applications of HSCs [69, 70]. Large-scale cell expansion requires sufficient medium containing essential nutrients and growth factors for the growing cell population. Medium flow also induces shear stress, so it is necessary to study whether shear stress affects HSC behavior. In addition, stromal cells at different regions in AGM lead to changes in the maintenance and differentiation of embryonic HSCs [71]. The biophysical interactions between embryonic HSCs and stromal cells in the AGM compartment regulate the development of embryonic HSCs [72]. These indicate that the activity of embryonic HSC requires the coordinated regulation of chemical signals, biological signals and physical signals from the AGM microenvironment.

### Biophysical microenvironment of BM niches

BM niches are the main site of adult HSC maintenance and regulation. Most adult HSCs are not directly exposed to the fluid environment in the BM, but some functional cells in the HSC niche may be in the fluid environment and will react the influence of fluid to HSCs through paracrine signals [41]. The fluid flow in the cavity of the bone produces shear stresses of 6–50 dynes/cm^2^. These shear stresses have been shown to affect bone cells and endothelial cells, thereby regulating the quiescence and circulation of HSC [73]. In BM, the fibronectin-rich endosteum region is stiff (40–50 kPa), while the laminin-rich perivascular region is soft (3 kPa) [74, 75]. Moreover, niche stiffness is not a static parameter, but rather a dynamical property during physiological processes. During the mobilization of HSCs from BM niches to the blood circulation, adrenaline stimulates osteoblasts to flatten and harden [45, 76]. The composition and molecular cross-linking in the ECM will change due to aging and disease, leading to matrix stiffening [77, 78]. Similarly, in the occurrence of diseases such as atherosclerosis and myelofibrosis, the hematopoietic tissue is hardened. Research has shown that both embryonic and adult stem cells are sensitive to substrate stiffness, including HSCs. By changing the E-modulus of the substrate, the differentiation of MSCs can be controlled [79, 80]. Our previous work has also shown that matrix stiffness regulates macrophage growth and development [81]. Besides matrix stiffness, matrix elasticity, and nanotopography of the matrix can also regulate the adhesion of HSCs [82]. In addition, mechanical loading is required for HSC differentiation. When organisms are exposed to some special environments, the biophysical microenvironments in vivo will also change [83].

### Biophysical microenvironment of extra-medullary HSC niches

When the BM microenvironment becomes unsatisfactory, extra-medullary hematopoiesis may occur in the liver or spleen [27]. Clearly, the physical microenvironment provided by the liver or spleen for HSCs is completely different from the BM niche. Taking stiffness as an example, the Young's modulus of the liver and spleen ranges from 4 to 7 kPa, which is comparable to the stiffness of blood vessels, but far from the Young's modulus of the endosteal region. Most adult HSCs are located in BM, but a small number of HSCs circulate in the body [84]. Blood vessels provide another important extra-medullary HSC niche. Although blood vessels have no hematopoietic function, they are an important site for HSC activity. The biophysical characteristics of these extra-medullary niches are obviously different from those of the BM niches. For example, the shear force, which is generated by blood flow in the blood vessels, is an important stimulating factor that cannot be ignored. In mice, when HSCs circulate via blood flow, the shear stress in some areas exceeds 600 dyne/cm^2^ [85].

### Changes in the biophysical microenvironmental caused by aging and disease

Aging will change the microenvironment of HSCs, resulting in the decline of HSC function. Individual HSCs can exhibit lineage bias, giving rise to myeloid-biased, lymphoid-biased, or more balanced differentiation, with the proportion of myeloid-biased HSCs increasing with age [86]. With the increase in age, the balance of long-term hematopoietic stem cells (LT-HSCs) in maintaining hematopoietic output is destroyed. LT-HSCs give rise to myeloid-biased, and myeloid leukemia eventually develops. Using single-cell RNA sequencing (scRNA-seq) [87], a previous study identified an age-related myeloid-biased subset and revealed important regulators of inflammatory myeloid bias, providing guidance for preventing aging-induced myeloid leukemia [88].

The vascular remodeling and changes in adrenergic signaling during aging influence the niche function. HSCs and their derivatives remodel the niche component. These events may cooperatively bias the fate of HSCs toward myeloid differentiation [89].

## Mechanosensors and mechanotransduction in HSCs

**Fig. 4:**
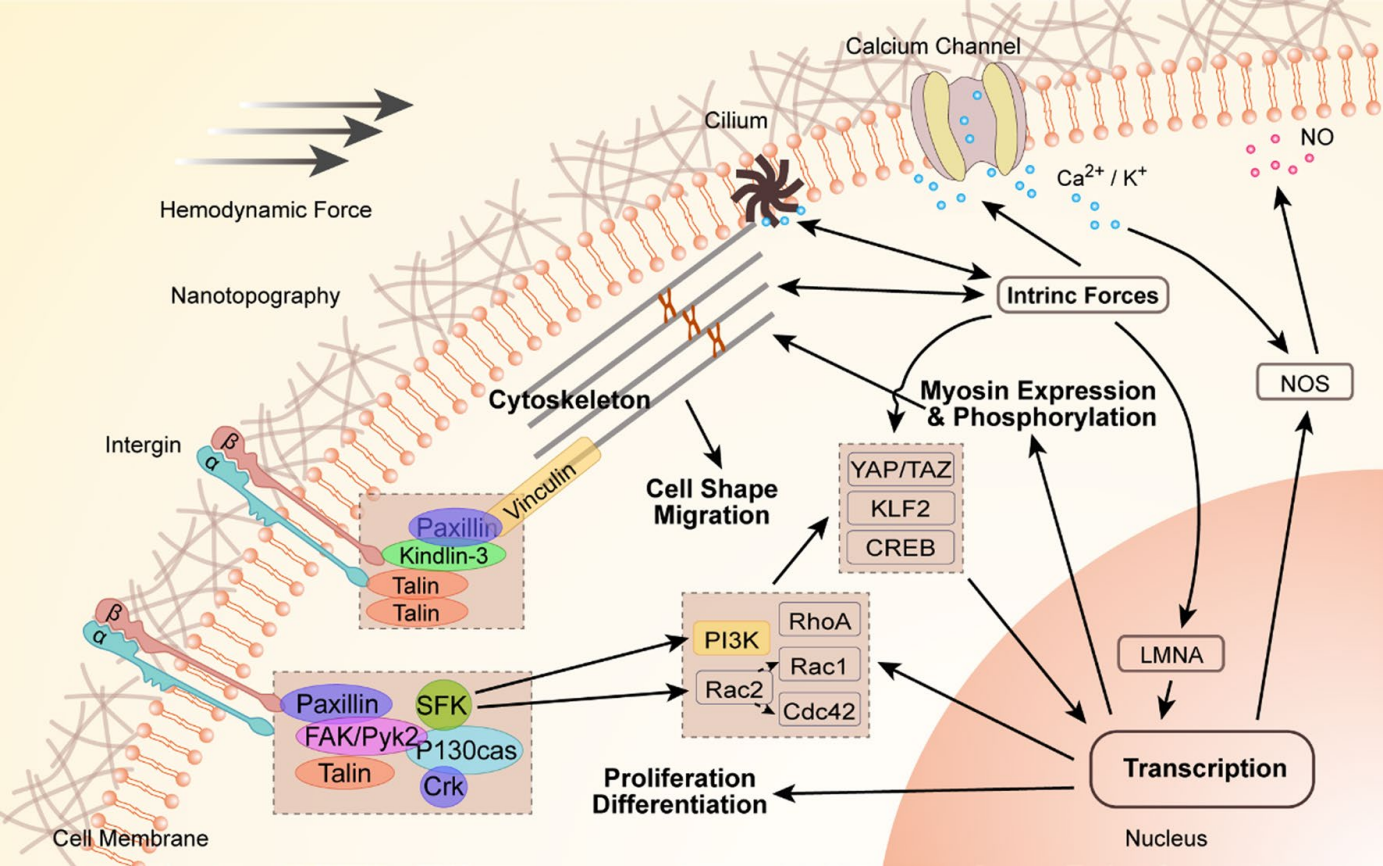
Mechanosensors and mechanotransduction of HSC. The drawing schematically depicts the mechanosensory units and molecules and highlights the molecules downstream of integrin that are expressed by HSPCs and/or play a role in HSPC biology. Biomechanical inputs from external loads directly stimulate mechanosensors such as mechanically gated ion channels, adhesion receptor-ligand bonds, cytoskeleton, and primary cilia. Intrinsic forces are generated under environmental mechanical constraints, and then transmit to neighboring cells through junctional interfaces, and consequently elicit cellular mechanoresponses. Besides, intrinsic forces can directly pass on to the nucleus through lamin A/C (LMNA), affecting chromatin structure and thereby controlling epigenetic processes. Biomechanical cues cooperate with biochemical signals in mechanotransduction

The HSC senses the biomechanical signals from the HSC niche through mechanosensors, thereby generating intrinsic forces and triggering a series of mechanotransduction. Finally, biomechanical signals will affect the cytoskeleton and chromatin structure, thus guiding cell behavior (Fig. 4).

### Mechanosensors

The signals generated by supporting cells and ECM are necessary for the maintenance and regulation of HSCs [90, 91]. The mechanosensors formed by cell–cell and cell–ECM interactions, such as adhesion receptor–ligand bonds, the cytoskeleton, mechanically gated ion channels, and primary cilia, enable HSCs to autonomously sense and react to mechanical cues in niches [36].

#### Integrin-mediated adhesion

Cell adhesion molecules (CAMs) mediate cell–cell and cell–ECM connections. The domain formed by the connection initiates intracellular signal transduction or interacts with the cell cytoskeleton. CAMs include integrins, cadherins, selectins, and the immunoglobulin superfamily [92, 93]. Cadherin mediates intercellular adhesion, while integrin mediates cell–ECM adhesion and finally uses intrinsic forces to form focal adhesions (FAs) [74, 94, 95]. ECM proteins with Arg-Gly-Asp integrin recognition motifs, such as fibronectin, laminin, and collagen, can connect with integrin, thus enabling cells to sense mechanical signals [73]. The activated integrin will bind and activate kindlins and talins. In addition, vinculin combines with talin to promote the enrichment of multiple activated integrins. Vinculin also transmits signals to the cytoskeleton by combining its tail domain with actin [96, 97].

In addition to transmitting the signal to the cytoskeleton, integrin also transmits the signal into the cell through the intracellular multi-protein complex [98]. In this process, paxillin, focal adhesion kinase (FAK), Pyk2, Crk, and P130CAS are induced to phosphorylate. These key elements play a role in the mechanotransduction of HSCs. FAK and Pyk2 interact with talin and paxillin and are involved in the activation of paxillin and guanine nucleotide exchange factors [99, 100]. When the adaptor molecule P130cas, as a mechanosensor located at the downstream of integrins, is phosphorylated, it becomes the substrate for the interaction of kinases of the Src family [101]. In addition, the Src family kinases (SFKs) are quickly activated. The SFKs can directly bind with integrin and can also connect with FAK. SFKs have an effect on the mobilization of HSCs from the BM to the circulation and play a role in HSCT [102, 103]. Phosphatidylinositol-4,5-bisphosphate3-kinase (PI3K) activated by SFKs can regulate HSC adherence and motility [76]. The HSCs can sense the force from the ECM and can apply traction force to the ECM in return. HSCs can secrete matrix components or proteases to regulate the ECM, thus enhancing or eliminating the adhesion interactions between HSCs and the ECM [104, 105]. ECM remodeling proteins change the niche microenvironment, thus regulating the quiescence, mobilization, and hematopoiesis of HSCs [106–108].

#### Intrinsic forces generated by the cytoskeleton

The cytoskeleton is the communication hub between the cell and the external biophysical microenvironment. Through the changes of the cytoskeleton, HSCs can sense, transmit, and generate force [109–111]. It has been shown that myosin IIA in HSCs is regulated by matrix stiffness. The activity of myosin IIA is enhanced on stiff matrices, whereas it is decreased on soft matrices [112, 113]. When the cytoskeleton or the transmembrane adhesion receptors connected with the cytoskeleton are stimulated by biomechanical stimuli, the cytoskeleton is remodeled and the cytoskeleton tension is rearranged, thus generating intrinsic forces [114, 115]. Through a component of nuclear lamina proteins, laminin A/C, the intrinsic force can be directly transmitted to the nucleus, thus modifying the chromatin structure and controlling epigenetic transcription [116]. In Ptpn21 deletion, HSCs, by dephosphorylating Spetin1 in cells, damages the stability of the cytoskeleton, reducing the decrease in HSC stiffness and increasing the physical deformation ability of HSC, thus weakening the quiescence and hematopoietic reconstitution capabilities of HSCs [117]. Ptpn21-deleted leukemic cells also showed a decrease in mechanical rigidity and an increase in cell deformability. These studies support the concept that the cytoskeleton is a hub of communication in mechanotransduction [118].

#### Mechanically gated ion channel

Cationic stretch-activated channels can sense mechanical forces as well as intrinsic forces and are permeable to Ca^2+^ as the second messenger [134–137]. The blocker, activator, or modulator of Na^+^ /K^+^-channels can regulate the fate of HSCs [64]. Ca^2+^ can regulate the activity of eNOS and stimulate the release of nitric oxide (NO). NO is a necessary regulator for HSC functions [119]. The depletion of NO in HSCs leads to the transformation of HSCs from differentiation to proliferation [120].

#### Primary cilia

Increasing evidence indicates that the primary cilia in almost all human blood and BM cells (97–99%) may be a communication hub for signal transduction. Because of the abundant calcium channels and receptors in its membrane, cilia have the ability to sense and transmit microenvironmental mechanical and chemical stimuli [121, 122]. The mechanical signals transmitted by the primary cilia are required for the osteogenic response and proliferation of human MSCs, and thus contribute to the maintenance of the essential components of BM niches that support HSCs [123]. In addition, vascular ECs sense the fluid flow signals through the primary cilia and regulate the biosynthesis of NO [124, 125]. However, the further influence of the NO released mediated by the primary cilia on the outputs of HSCs within vascular niches remains unclear.

### Mechanoresponsive transcription factor

On and inside the cell membrane, changes in cytoskeletal remodeling and protein recruitment are the first step of mechanical signal inputting. This step introduces the downstream mechanotransductive effects, thus stimulating the changes in the cytoplasmic localization of molecules and ultimately stimulating transcriptional effects. Yes-associated protein (YAP) and transcriptional co-activator with PDZ-binding motif (TAZ) are two transcriptional cofactors that shuttle between the nucleus and the cytoplasm. They can transmit signals triggered by biomechanics to the nucleus to affect gene transcription [109, 110]. The activity of YAP/TAZ is limited to cells experiencing biomechanical stresses, and its localization and degradation are regulated by the Hippo pathway [110]. YAP is detectable at low levels only in murine long-term HSCs, but not in murine short-term HSCs or Lin^+^ hematopoietic lineages. In addition, the nuclear skeleton proteins laminin A and laminin B are important elements involved in biomechanical signal transmission to the nucleus. These proteins have been confirmed to be biomechanosensitive and play an important role in HSC transmigration [126]. KLF2 is an important biomechanically activated transcription factor and a key medium for HSC production induced by blood flow [127, 128]. cAMP response element-binding protein (CREB) is a downstream effector of fluid shear stress that has been demonstrated to affect the emergence of HSCs [129, 130].

### Complicated crosstalk under mechanical conditions

The above describes the mechanosensors and mechanotransduction in HSCs. Mechanosensors and mechanotransduction work together to determine the fate of HSCs. The cytoskeleton is closely related to YAP/TAZ. Studies have shown that the F-actin related protein can enhance the YAP nuclear translocation and can abrogate YAP/TAZ activity [131, 132]. Cdc42-Rho-GTPase promotes F-actin polymerization to enhance the nuclear retention of YAP [133]. Ciliary bending caused by biomechanical stimuli can induce cytoskeletal deformation and membrane stretching, thus initiating extracellular Ca^2+^ influx through calcium channels in the ciliary membrane [134, 135]. In short, these mechanosensors and mechanotransduction are highly interconnected rather than mutually exclusive.

Currently, previous studies have shown that HSC is mechanically sensitive, and some biomechanical sensing elements have been proposed, but the molecular mechanism of mechanotransduction needs further clarification.

## Engineering the biophysical niches for clinical applications

### Clinical significance of mimicking niche biophysical signals

Stem cell therapy, such as transplantation and tumor purging, is used to treat hematological diseases and malignant tumors [136]. HSCT was achieved in the 1950s [137]. The BM or HSCs extracted from autogenous or allogeneic grafts can be infused into patients after myeloablative treatment [74]. The main bottleneck of this treatment is the lack of sufficient HSC supply, because the number of stem cells from common sources such as the BM and umbilical cord blood is scarce [138]. In addition, there are also obstacles to the function of transplanted HSCs, which are mainly manifested in the low homing efficiency of HSCs transplanted into the BM cavity [139]. Therefore, how to effectively amplify HSCs in vitro is of great significance for clinical treatment.

The method of amplifying HSCs in vitro by referring to the natural microenvironment of HSCs is emerging. However, the common HSCs culture system only focuses on the provision of growth factors and cytokines, and rarely pays attention to the influence of biomechanical clues. Such systems can enhance the proliferation of HSPCs, but the proliferating HSCs have differentiated and lost their self-renewal ability, which has no clinical significance [140, 141]. Therefore, in order to reproduce the natural microenvironment of HSC, physical factors must be considered. Some designs with biomechanical clues help to realize the continuous expansion of HSCs while maintaining their ability to self-renew and differentiate [142, 143].

### Engineering HSC niches in vitro

How to reconstruct the unique and intricate microenvironment architecture and nanotopography of HSC niches in vivo and provide a variety of biophysical cues for HSCs and HSC-related accessory cells are important research directions of HSC culture in vitro [13, 37]. Through biomaterial technology, scaffolds with complex structures can be manufactured to provide biophysical clues for cells [144]. The methods of mimicking the BM niche in vitro and the evaluation of their advantages and disadvantages were summarized in our previous investigation [11].

BM bionic three-dimensional (3D) scaffold can better maintain and expand HSCs than traditional two-dimensional (2D) culture system [142]. Human umbilical cord blood HSCs proliferate more strongly in 3D scaffolds than in 2D conditions [145–149]. Similarly, compared with the standard 2D culture system, MSCs have a more significant positive effect on the proliferation of HSPC in the 3D PEG co-culture system [150]. This method of combining stromal cells with bioscaffolds mimics the BM microenvironment more effectively. Our group has investigated the regulation of HSC by matrix dimensionality. Compared to 2D cultures, HPCs within 3D systems generate a cluster of “3D-macrophages,” and 3D matrices enhance the communications between such 3D macrophages and other hematopoietic clusters based on bioinformatic analyses [151]. The proportion of Lin^−^ c-kit^+^ Sca1^+^ (LSK) cells in BM cells can be significantly increased by culture on stiff matrix [36]. In addition, although the adhesion of HSCs to material surfaces is not strong enough [152], many studies have found that nanotopography can indeed affect the behavior of HSCs. Since research found that nanotopography can affect the behavior of HSPC [152], the regulatory mechanism of nanotopography on HSCs has received extensive attention.

## Conclusion and perspective

The clinical bottleneck of HSCT is how to efficiently proliferate functional HSCs in vitro. Identifying the influencing factors during HSC development and grasping the underlying mechanisms are the key to understanding why HSCs have the abilities of self-renewal and versatility. The complex and exquisite HSC niche can provide physical, chemical, and biological stimuli to regulate HSC survival, maintenance, proliferation, and differentiation. When identifying the specific contribution of the microenvironment to HSC fate, all types of environmental stimuli acting on cells must be considered.

The study of biomechanical signals has been underestimated in research on HSC niches. This is a breakthrough complementary theory that improves and expands the currently known types of HSC regulatory signals. This review introduced the spatiotemporal heterogeneous biophysical microenvironment during HSC development, homeostasis, and malignancy, illustrated how these biophysical cues contribute to HSC behaviors, and discussed the possible mechanotransduction mechanisms from the extracellular microenvironment into cells. Comprehending the important functions of these biophysical regulatory factors will provide novel approaches for resolving clinical problems.

HSC maintenance and self-renewal are strictly regulated throughout the human lifetime to maintain blood system homeostasis. HSC niches are spatiotemporally heterogeneous during HSC development, homeostasis, and malignancy. In studying the specific contribution of the biophysical microenvironment to HSC fates, the heterogeneity of the composition and function of niches in different anatomical sites at different developmental stages should be emphasized. In addition, under some special environments such as weightlessness and high-altitude hypoxia, abnormal physiological processes such as obesity and malignancy will cause changes in the microenvironment of the niche, which in turn affects the fate of HSCs. This is also a direction for future research.

HSC can sense and transmit the biophysical signals from HSC niche, so as to guide the behavior of HSC. However, most studies only describe the phenomenon that HSC has biomechanical sensitivity, without clarifying the intrinsic molecular mechanism, and some key problems have not been solved: Whether HSC has the same sense and transmission mode for different biomechanical signals, whether the feedback of HSC to biomechanical signals is short term or long term, and whether the complex process of HSCs regulated by biomechanical signal, biological signal, and chemical signal has series connection. These are still hot topics to be studied in the future.

Burgeoning experimental techniques have also facilitated HSC research, such as single-cell sequencing [87], high spatiotemporal resolution imaging, CyTOF [153], and bioengineering. Advances in these technologies will allow researchers to elucidate the mechanisms through which the physical microenvironment regulates HSC fate, and thus it is conceivable that harnessing these biophysical cues as master regulators for HSC fate regulation could be exploited for artificial niches and therapeutic gain.

## Data Availability

Data sharing is not applicable to this article as no datasets were generated during the current study. The images depicted in Figs. 1, 2, 3 and 4 are drawn by ourselves.

## Abbreviations

AGM: Aorto-gonad-mesonephros
BM: Bone marrow
CB: Cord blood
CyTOF: Cytometry by time of flight
ECM: Extracellular matrix
ECs: Endothelial cells
EHT: Endothelial-to-hematopoietic transition
EMH: Extra-medullary hematopoiesis
FAK: Focal adhesion kinase
FAs: Focal adhesions
HSC: Hematopoietic stem cell
HSCT: Hematopoietic stem cell transplantation
HSPC: Hematopoietic stem and progenitor cell
LINC: Linker of nucleoskeleton and cytoskeleton
LMNA: Lamin A/C
LSK: Lin^−^ c-kit^+^ Sca1^+^
LT-HSC: Long-term hematopoietic stem cell
MSC: Mesenchymal stromal cells
NK: Natural killer
NO: Nitric oxide
PAM: Polyacrylamide
PI3K: Phosphatidylinositol-4,5-bisphosphate3-kinase
scRNA-seq: Single-cell RNA sequencing
SFKs: Src family kinases
TAZ: Transcriptional co-activator with PDZ-binding motif
TF: Transcription factors
UCB: Umbilical cord blood
WSS: Wall shear stress
YAP: Transcriptional cofactors Yes-associated protein
